# Neuroprotective potential of carvacrol: restoration of oxidative balance and mitigation of brain injury markers in isoproterenol-induced rats

**DOI:** 10.1007/s11011-025-01634-6

**Published:** 2025-05-23

**Authors:** Betül Bağcı, Şeyma Aydın, Elif Dalkılınç, Selim Çomaklı, Sefa Küçükler, Selçuk Özdemir

**Affiliations:** 1 Department of Molecular Biology and Genetics, Faculty of Science, Atatürk University, Erzurum, Türkiye; 2 Department of Genetics, Faculty of Veterinary Medicine, Atatürk University, Erzurum, Türkiye; 3 Department of Biochemistry, Faculty of Veterinary Medicine, Atatürk University, Erzurum, Türkiye; 4 Department of Pathology, Faculty of Veterinary Medicine, Atatürk University, Erzurum, Türkiye

**Keywords:** Oxidative stress, Neuroinflammation, Carvacrol, Mitochondrial dysfunction, Brain injury markers

## Abstract

This research investigated the protective properties of Carvacrol (CVC) against Isoproterenol (ISO)-induced oxidative stress, neuroinflammation, and mitochondrial dysfunction in rats. The findings showed that CVC treatment did not significantly modify baseline oxidative stress levels in healthy rats but successfully alleviated ISO-induced oxidative damage by augmenting antioxidant enzyme activity and diminishing lipid peroxidation, as demonstrated by a reduction in MDA levels. These findings indicate that CVC can reinstate antioxidant capability and reduce oxidative damage. Concerning neuroinflammation, ISO therapy markedly increased the expression of pro-inflammatory markers, including TNF-α, IL-1β, c-Fos, BDNF, Nfl, and GFP, signifying a robust inflammatory and damage response. The injection of CVC following ISO exposure markedly decreased the expression of these markers, suggesting that CVC may exert a neuroprotective effect by regulating the inflammatory response and mitigating neuronal and glial damage. CVC demonstrated a notable protective effect on mitochondrial integrity, evidenced by the decreased mRNA expression of mitochondrial damage markers, including NSE, s100B, CALP1, and CALM1 in the CVC-treated groups, showing that CVC mitigates mitochondrial dysfunction. The analysis revealed no significant alterations in the expression levels of Aβ40, pTau181, and tTau across all groups, indicating that these biomarkers were not substantially influenced by CVC treatment under the study’s conditions. However, β-amyloid accumulation varied significantly between groups, highlighting the need for further research to explore CVC’s potential implications in amyloid-related diseases. These findings endorse CVC’s neuroprotective efficacy and therapeutic potential in neurological disorders associated with oxidative stress, inflammation, and mitochondrial impairment.

**Graphical abstract:**

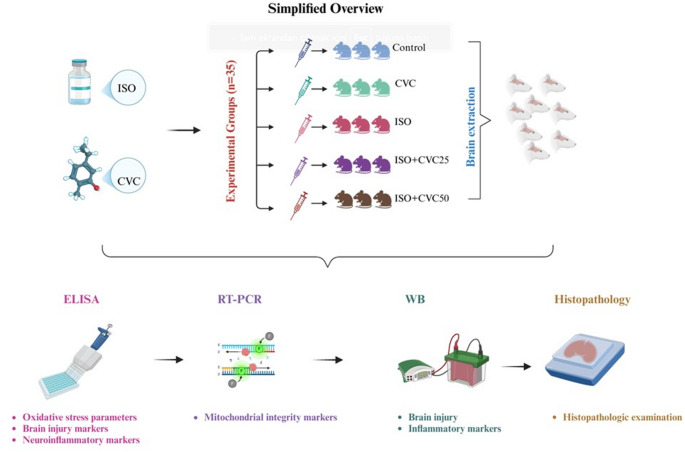

**Supplementary Information:**

The online version contains supplementary material available at 10.1007/s11011-025-01634-6.

## Introduction

Isoproterenol (ISO), a synthetic sympathomimetic amine (catecholamine), was identified in 1959 (D’Ambrosi and Amin 2018). It is structurally analogous to adrenaline, a naturally occurring catecholamine in the body, and replicates many of its physiological actions. Adrenaline activates alpha-adrenergic receptors to elicit various biological responses, whereas ISO predominantly functions as a beta-adrenergic agonist, exerting its most significant effects on beta-1 and beta-2 adrenergic receptors, with a comparatively weak influence on alpha receptors (Kahn et al. 1969). This selective action enables ISO to elicit specific effects, including elevated heart rate, bronchodilation, and vasodilation. These characteristics render ISO beneficial for treating ailments such as bradycardia and respiratory disorders like asthma. Owing to its structural and physiological parallels with adrenaline, ISO has emerged as a significant instrument in pharmacological research and the formulation of novel therapeutic techniques.

High dosages of ISO can cause myocardial ischemia (Keihanian et al. 2021; Lan et al. 2022). This ischemia arises from ISO’s strong positive inotropic and positive chronotropic effects on the heart. These effects elevate myocardial oxygen demand, diminish diastolic filling pressure and coronary perfusion pressure, ultimately resulting in ischemic alterations in the myocardium. The resultant changes resemble those seen after a myocardial infarction in people (Tiwari et al. 2009). If myocardial ischemia persists for an extended period, it can lead to a reduced oxygen supply throughout the body, potentially causing brain damage due to insufficient oxygenation. However, there is limited information and clarity regarding the exact relationship between prolonged myocardial ischemia and its direct effects on the brain.

Hypoxic-ischemic encephalopathy (HIE) is a type of brain injury caused by hypoxia and diminished cerebral blood flow, resulting in mortality and enduring neurological consequences (Dumbuya et al. 2021; Garcia-Alix et al. 2024). The manifestation of HIE is an ongoing process that results in neuronal cell death within hours following the first insult. In the initial phase, ischemia and hypoxia cause a depletion of cellular energy, leading to oxidative metabolic failure, accumulation of excitotoxic chemicals, cell death, intracellular Ca^2+^ overload, and edema. The subsequent phase is the latent phase, during which the body reinstates respiration and cerebral circulation; nevertheless, this process results in reperfusion injury and oxidative stress. Neuroinflammation and mitochondrial malfunction arise during this period, activating pro-apoptotic signaling pathways (Cánovas-Ahedo and Alonso-Alconada 2019; Andelius et al. 2020; Ranjan and Gulati 2023). This leads to secondary energy failure, termed delayed neuronal death, which exacerbates cell death, excitotoxicity, and the delayed maturation of oligodendrocytes. Chronic inflammation and epigenetic modifications may result in enduring cerebral damage, which can last for weeks, months, or even years, ultimately inflicting considerable detriment to the body. The pathogenesis of hypoxic-ischemic encephalopathy (HIE) primarily encompasses mechanisms including hypoxia-ischemia, reperfusion injury, inflammation, oxidative stress, mitochondrial dysfunction, excitotoxicity, ferroptosis, endoplasmic reticulum stress (ERS), cellular swelling, impaired maturation, and apoptosis (Molloy et al. 2024).

Since antiquity, plant-derived products and their derivatives have been esteemed as sources of medicinal substances. Currently, essential oils and extracts from diverse plant sources are being investigated for their potential as antioxidant agents. Thyme is a plant that has historically served as both a spice and a medicinal remedy. Carvacrol (CVC), a monoterpene phenol characterized by a structure including two connected isoprene units, was initially discovered in thyme and subsequently recognized in the essential oils of various other aromatic plants (Can Baser 2008; Gandova et al. 2023; Mączka et al. 2023; Chaachouay and Zidane 2024). Currently, CVC is extensively employed in the food and cosmetics sectors. Multiple studies have emphasized its antifungal, antibacterial, and antioxidant characteristics (Imran et al. 2022). Furthermore, CVC has several advantageous effects, such as antimutagenic, antigenotoxic, antispasmodic, angiogenic, antiparasitic, insecticidal, and antihepatotoxic properties. In addition to its many beneficial effects, CVC also possesses regenerative properties. Numerous studies have further supported its antioxidant activity. CVC is considered an effective antioxidant due to the presence of a hydroxyl group (OH-) in its chemical structure. When examining the relationship between thyme’s antioxidant properties and its chemical compounds, it has been demonstrated that the antioxidant activity is directly related to the concentration of antioxidants present (Can Baser 2008; Imran et al. 2022; Gandova et al. 2023; Mączka et al. 2023; Chroho et al. 2024; Chaachouay and Zidane 2024). Research has also shown that CVC exerts neuroprotective effects in both mice and rats. The neuroprotective potential of CVC is primarily attributed to its ability to reduce lipid peroxidation and prevent cell death (Li et al. 2016; Dati et al. 2017; Cicalău et al. 2021; Shah et al. 2024).

The S100B protein is part of a multigenic family of low molecular weight (9–13 kD) calcium-binding S100 proteins. S100B is predominantly found in glial cells of the central nervous system, particularly in astrocytes (Donato 1986; Heizmann et al. 2002). Enolases are glycolytic enzymes that exist as a sequence of dimeric isoenzymes composed of three immunologically different subunits: the α, β, and γ chains. The isoforms γ γ and α γ are confined to neurons, peripheral neuroendocrine tissue, and malignancies associated with the amine precursor uptake and degradation system (Cooper 1994). Calmodulin (CaM) is a versatile calcium ion sensor protein that mediates a significant portion of calcium signaling. CaM is a dumbbell-shaped protein with two structurally similar globular domains, each featuring a pair of EF-hand calcium-binding motifs, interconnected by an elongated flexible central helix (Ikura and Ames 2006).

In this study, we characterized the extent of brain damage induced by ISO administration, highlighting the underlying mechanisms of neuronal injury. Furthermore, we examined the potential therapeutic effects of CVC in mitigating the neuronal damage caused by ISO, assessing its ability to counteract oxidative stress, inflammation, and cellular damage within the brain.

## Material and method

### Reagents

Isoproteranol was purchased from Thermo Scientific (Cat. No: 437210050, purity: 98%, Waltham, MA, United States). Carvacrol was obtained from Sigma-Aldrich (Cat. No: W224502, purity ≥ 98%, St. Louis, MO, United States).

### Experimental design

This study utilized 35 male Sprague Dawley rats, each weighing between 250 and 300 g. All animals were housed in a regulated environment with a constant temperature of 24 ± 2 °C and humidity levels of 40–80%, maintained on a 12-hour light/dark cycle. The rats were granted unlimited access to food and water during the research. A week of acclimatization time was permitted for adaptation to the environment before the commencement of the experiment. The research was performed at the Atatürk University Medical Experimental Research Center.

The experiment had five distinct groups, each consisting of seven male Sprague Dawley rats. The groups were structured as outlined below:

- Group 1: Control: Rats received oral administration of distilled water for 14 days.
- Group 2: Isoproterenol (ISO): Rats were administered ISO (100 mg/kg/bw), dissolved in saline, via subcutaneous injection at 24-hour intervals for two consecutive days (Days 13 and 14) (Kumar et al. 2017).
- Group 3: Carvacrol (CVC): Rats received an oral administration of CVC at a dosage of 50 mg/kg/bw for 14 days (Kandemir et al. 2021).
- Group 4: Isoproterenol + Carvacrol 25 (ISO + CVC25): Rats were administered CVC orally at a dosage of 25 mg/kg/bw for 14 days. On Days 13 and 14, ISO (100 mg/kg/bw), diluted in saline, was delivered subcutaneously at 24-hour intervals.
- Group 5: Isoproterenol + Carvacrol 50 (ISO + CVC50): Rats received CVC orally at a dosage of 50 mg/kg/bw for 14 days. On Days 13 and 14, ISO (100 mg/kg), diluted in saline, was delivered subcutaneously at 24-hour intervals.

At the end of the trial, the rats were euthanized using mild sevoflurane anesthesia. Brain tissues were collected and preserved at −80 °C, while some were preserved in a 10% formaldehyde solution. Biochemical, histological, and Real-Time PCR methodologies were utilized on brain tissue specimens to assess the putative neuroprotective properties of CVC against ISO-induced brain injury.

### Assessment of oxidative stress

We measured the activities of antioxidant enzymes (Superoxide Dismutase - SOD, Catalase - CAT, and Glutathione Peroxidase - GPx), along with the levels of Glutathione (GSH) and Malondialdehyde (MDA) in the brains of rats using ELISA. Specific ELISA kits were employed for each parameter: Rat Superoxide Dismutase (SOD) ELISA Kit (Cat No: MBS036924, Detection Range: 12.5 U/ml − 400 U/ml), Rat Catalase (CAT) ELISA Kit (Cat No: MBS2600683, Detection Range: 1.56 ng/ml − 100 ng/ml), Rat Glutathione (GSH) ELISA Kit (Cat No: MBS9712516, Detection Range: 50 ng/L − 800 ng/L), Rat Glutathione Peroxidase (GPx) ELISA Kit (Cat No: MBS744364, Detection Range: 0 ng/mL − 25 ng/mL), and Rat Malondialdehyde (MDA) ELISA Kit (Cat No: MBS727531, Detection Range: 0 ng/mL − 100 ng/mL). ELISA measurements were performed following the specific protocols outlined in the kit instructions.

### Assessment of biomarkers associated with brain injury

The brain injury caused by ISO was evaluated by quantifying various biomarkers, including TNF-alpha, IL-1β, GFAP, Nfl, BDNF, and c-Fos, by ELISA methodology. The following markers were chosen because of their correlation with neuroinflammation, neuronal damage, and neuronal activity. Specific ELISA kits for each biomarker were employed to measure their quantities in brain tissue samples, adhering to the manufacturer’s guidelines. Rat Tumor necrosis factor alpha (TNF-alpha) ELISA Kit, Cat No: MBS282960, Detection Range: 6.25 pg/mL − 400 pg/mL; Rat Interleukin 1 Beta (IL-1β) ELISA Kit Cat No: MBS265868, Detection Range: 15.6 pg/mL- 1000 pg/mL; Rat c-Fos ELISA Kit, Cat No: MBS729725, Detection Range: 0 ng/mL-10 ng/mL; Rat BDNF ELISA Kit Cat No: MBS355345, Detection Range: 31.2 pg/ml-2000 pg/ml; Rat Neurofilament-Light Chain (Nfl) ELISA Kit Cat No: MBS9399608, Detection Range: 3.12ng/ml-100ng/ml; Rat GFAP ELISA Kit, Cat No: MBS2505953, Detection Range: 0.31 ng/mL-20 ng/mL.

### Quantitative measurement of Amyloid Beta 40, total Tau and pTau181 protein levels by ELISA

Amyloid Beta 40, total Tau, and pTau181 protein concentrations were quantitatively assessed utilizing commercial ELISA kits. Brain samples were homogenized, preserved at −80 °C, and subsequently examined. Each sample was introduced to ELISA plates with standards and other samples, followed by a 2-hour incubation period. Subsequently, washing was conducted, followed by the addition of secondary antibody/enzyme conjugates, which were incubated for one hour. The reaction was initiated with TMB substrate and concluded by the addition of termination solution. Optical density (OD) values were measured at 450 nm, and protein concentrations were determined using the standard curve (Rat Amyloid beta Protein 40 ELISA Kit Cat No: MBS727047, Detection range: 0 pg/mL-500 pg/mL; Rat total Tau Proteins (ttau) ELISA Kit Cat No: MBS1600235 Detection Range: 5ng/L − 1800ng/L; Rat Phosphorylated Tau 181 (pTau-181) ELISA Kit Cat No: MBS9905658, Detection Range: 6.25pg/ml-200pg/ml).

### Total RNA isolation

Tissue samples (50 mg) from experimental groups were combined with 1 ml of TRIzol-LS (Qiazol, Qiagen, Germany) and incubated at ambient temperature for 5 min. The materials were subsequently centrifuged at 12,000 x g at 4 °C for 15 min. The supernatant was transferred to a fresh Eppendorf tube, and 500 µl of chloroform was included. The solution was vortexed for one minute and subsequently centrifuged at 12,000 x g at 4 °C for fifteen minutes. The supernatant was subsequently transferred to a new Eppendorf tube, and 200 µl of isopropanol was included. The solution was centrifuged at 12,000 x g at 4 °C for 10 min. Following the removal of the supernatant, 500 µl of 75% ethanol was introduced to the RNA pellet, which was subsequently centrifuged at 7,500 x g at 4 °C for 10 min. After the removal of ethanol, the RNA pellet was reconstituted in a suitable volume of RNase-free, DEPC-treated water. The purity and concentration of the extracted RNA were assessed using absorbance measurements at 260–280 nm utilizing a spectrophotometer. 1 µg of RNA, measured using 260 nm absorbance, was applied to a 1.5% agarose gel to evaluate the integrity and amount of the RNA.

### cDNA synthesis

DNase I (Thermo Scientific) was employed to remove DNA contamination from the isolated total RNA samples. The application was executed following the manufacturer’s methodology. Subsequently, 2–5 µg of these RNA samples were transcribed into cDNA utilizing the miScript Reverse Transcription Kit (Qiagen) in accordance with the supplied instructions. The purity and concentration of the synthesized cDNA were assessed using absorbance measurements at 260–280 nm with a spectrophotometer, and the cDNA was diluted in equivalent ratios. The cDNA was subsequently preserved at −20 °C for application in Real-Time PCR analyses.

### Real-time PCR

RT-PCR was performed using the Qiagen Rotor Gene HRM-5 device to measure mRNA transcript levels of Neuron-Specific Enolase (NSE), S100 calcium binding protein B (S100B), Calbindin (CALB1), Calmodulin (CALM1), mitochondrial dynamin like GTPase (Opa1), dynamin 1-like (Dnm1 l), and cardiolipin synthase 1 (Crls1) genes. Glyceraldehyde-3-phosphate dehydrogenase (GAPDH) gene was used as internal controls. The master mix for the real-time PCR experiments consisted of Syber Green 2X Rox Dye Master mix (Qiagen), forward and reverse primers designed for the genes, cDNAs as templates, and nuclease-free water. After preparing the master mixes, the samples were analyzed in triplicate on the real-time device, and the expression levels of the relevant genes were determined by calculating the Ct/Cq values using the 2^(-ΔΔCt) method (Livak and Schmittgen 2001).

### Western blot analysis

50 mg of brain tissue was homogenized with the addition of 500 µL of Radioimmunoprecipitation Assay (RIPA) buffer (Cat. No.: sc-24948) while kept on ice. Homogenized samples underwent centrifugation at 12,000 × g for 15 min at 4 °C to isolate cell debris. The resultant supernatant served as the protein isolate. Protein concentration was determined using the Pierce™ BCA Protein Assay Kit (Rockford, IL, USA), and samples were standardized to ensure equal protein quantities. A 12% polyacrylamide gel was prepared, and 25 µg of protein were combined with a sample buffer and loaded onto the gel. Protein samples were subjected to a voltage of 120–150 V for a duration of 1 to 1.5 h for separation. After the separation step, proteins were transferred to nitrocellulose membranes using the Trans-Blot^®^ Turbo™ Transfer System (Bio-Rad). The membrane was incubated with 5% skim milk powder for 1 h at room temperature to prevent non-specific binding. Then, primary antibodies—Amyloid Beta (Santa Cruz, Cat. No.: sc-28365, RRID: AB_626669, Dilution Rate: 1:1000), IL-1β (Santa Cruz, Cat. No.: sc-52012, RRID: AB_629741, Dilution Rate: 1:1000), and GAPDH (Santa Cruz, Cat. No.: sc-32233, RRID: AB_627679, Dilution Rate: 1:500) —were applied to the membrane and incubated overnight at 4 °C. After incubation with primary antibodies, the membranes underwent three washes, each lasting 10 min, using TBS-T (Tris-buffered saline containing 0.1% Tween 20). Then, membranes were incubated with a secondary antibody (Santa Cruz, Cat. No.: sc-2005, RRID: AB_631736 Dilution Rate: 1:5000) for an hour at room temperature. Following three additional washes with TBS-T buffer, protein bands were visualized using the Clarity™ Western ECL (Enhanced Chemiluminescence) substrate (Bio-Rad). Imaging was performed with the ChemiDoc™ MP imaging system (Bio-Rad), and band intensities were analyzed using ImageJ software.

### Histopathological examination

At the end of the experiment, the brains of the animals were removed, and some were preserved in a 10% formaldehyde solution for 72 h to allow fixation. After fixation, the trimmed tissues were placed in tissue cassettes and washed under running tap water to remove any residual formaldehyde. The tissues were then processed through routine histological procedures, including gradual dehydration with increasing concentrations of alcohol, clearing with xylene, and embedding in paraffin using an automatic embedding machine. Sections, 5 μm thick, were cut from the paraffin blocks using a microtome (RM2255, Leica) and stained with hematoxylin and eosin. The staining protocol was as follows: Tissue sections were deparaffinized by incubating for 1 h at 57 ºC, followed by 5 min each in xylene I and xylene II solutions. Rehydration was performed by immersing the sections for 3 min each in gradually decreasing concentrations of alcohol (100%, 96%, 90%, 80%, 70%, and 50%), followed by 5 min in distilled water. The sections were immersed in a hematoxylin solution for 2 min and rinsed in distilled water for 1 min. Eosin staining was performed by immersing the sections in eosin solution for 10–30 s. Dehydration was carried out using 95% alcohol (twice) and 100% alcohol (twice) for 30 s each. The alcohol was removed using xylene (twice). One or two drops of Entellan were added to the sections, which were then covered with a coverslip. The prepared sections were examined under a light microscope (Leica DM750, Flexicam i5 camera). Different areas of the cortical and hippocampal regions were analyzed under a 40x objective lens for histopathological evaluation. Scoring was performed based on the observed findings using the following criteria: [0] No histopathological findings, [1] Mild findings, [2] Moderate findings, [3] Severe findings.

### Statistical analysis

Statistical analysis was performed utilizing both the R programming language and GraphPad software. The GraphPad software was employed to generate all figures, guaranteeing a clear and precise depiction of the experimental data. The Kolmogorov-Smirnov test was utilized to evaluate the data distribution and ascertain if it adhered to a parametric or non-parametric distribution. The test findings indicated that the data did not satisfy the assumptions necessary for parametric testing, hence suggesting a non-parametric distribution.

The Kruskal-Wallis test, succeeded by Dunn’s post hoc test, was utilized to compare group differences. The Kruskal-Wallis test is a non-parametric technique appropriate for examining differences among numerous independent groups when the data does not follow a normal distribution. The Dunn’s test was employed as a post hoc analysis to ascertain particular group differences subsequent to a significant Kruskal-Wallis outcome. This method guarantees rigorous statistical analysis and precise interpretation of the results, taking into account the non-parametric characteristics of the measurements derived from both ELISA and RT-PCR studies.

The Kruskal-Wallis test was employed to compare histopathological findings across multiple groups, and for pairwise comparisons between two groups, the Mann-Whitney U test was applied. A p-value of less than 0.05 (*p* < 0.05) was considered statistically significant.

## Results

### Protective effects of CVC on oxidative stress: amelioration of ISO-induced alterations

**Fig. 1:**
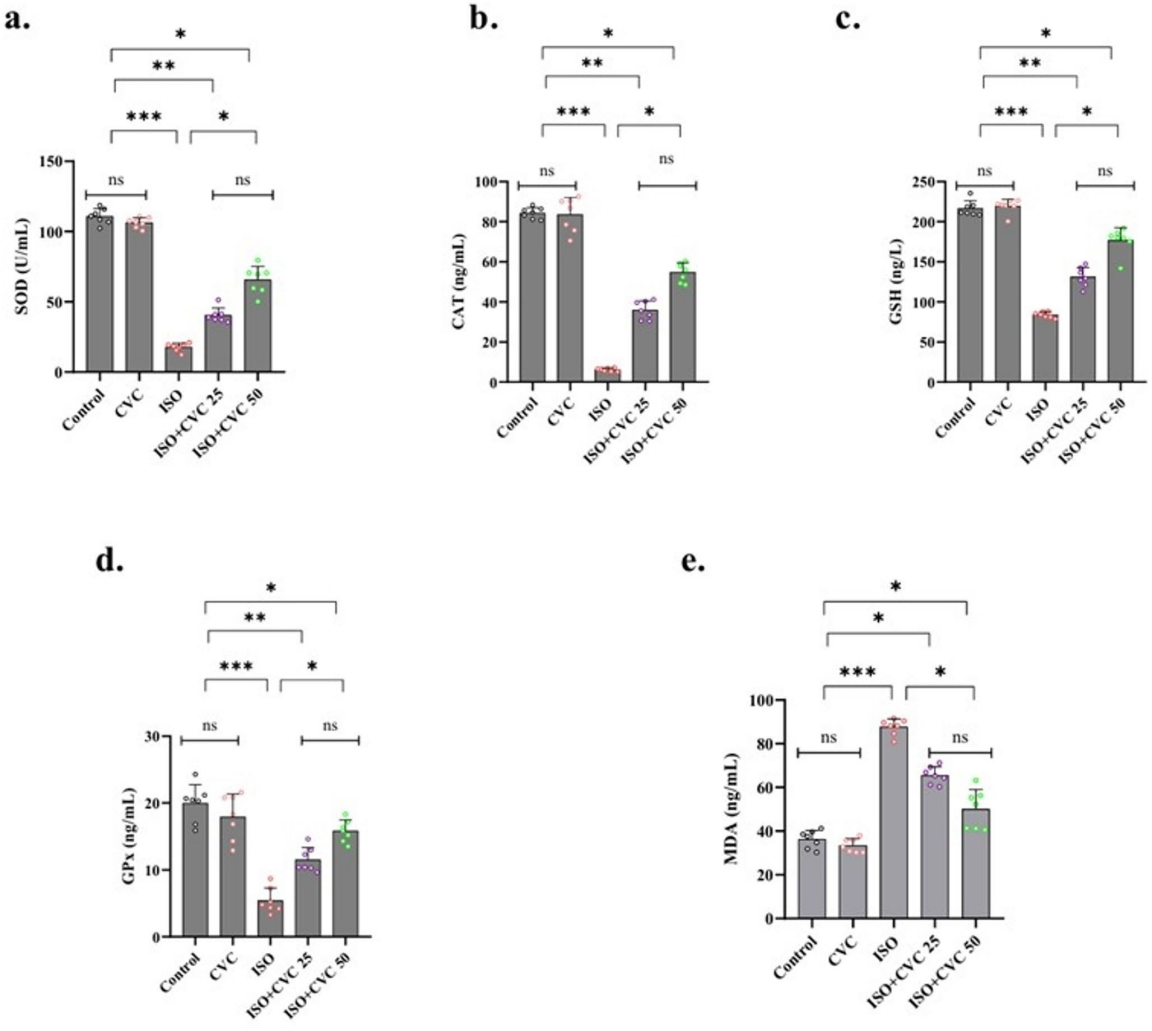
Effects of Carvacrol (CVC) on Oxidative Stress Markers in ISO-Induced Rats. (**a**-**d**) Antioxidant enzyme activity levels, including superoxide dismutase (SOD), catalase (CAT), glutathione (GSH), and glutathione peroxidase (GPx), were measured across the experimental groups. No significant changes were observed between the control and CVC-only groups (*p* > 0.05). The ISO group exhibited a significant reduction in these antioxidant enzymes compared to the control group (*p* < 0.01), indicating compromised antioxidant defense. Post-ISO administration of CVC significantly restored enzyme levels, reflecting an enhanced antioxidant capacity (*p* < 0.01). (**e**) Malondialdehyde (MDA) levels, an indicator of lipid peroxidation, were significantly elevated in the ISO group compared to the control (*p* < 0.0001), confirming ISO-induced oxidative damage. CVC treatment following ISO exposure significantly reduced MDA levels, indicating a decrease in lipid peroxidation and oxidative injury (*p* < 0.01)

The analysis of oxidative stress markers showed no statistically significant variations between the control group and the CVC-treated group (*p* > 0.05), suggesting that CVC administration alone did not modify baseline oxidative stress levels. In the ISO-induced group, there was a significant reduction in the levels of essential antioxidant enzymes, including SOD, CAT, GSH, and GPx (*p* < 0.01). This decline underscores a weakened antioxidant defense mechanism under ISO-induced oxidative stress conditions (Fig. 1a-d).

Furthermore, MDA, an indicator of lipid peroxidation and oxidative damage, exhibited a substantial elevation in the ISO group relative to the control (*p* < 0.0001), thereby substantiating the oxidative stress elicited by ISO administration (Fig. 1e).

The groups administered CVC post-ISO exhibited a notable enhancement in oxidative stress metrics. Specifically, levels of SOD, CAT, GSH, and GPx were considerably increased, indicating a restoration of antioxidant capacity (*p* < 0.01). Simultaneously, MDA levels were markedly diminished in these groups (*p* < 0.01), indicating a reduction in lipid peroxidation and oxidative injury (Fig. 1a-e).

These findings collectively indicate that CVC exerts a protective effect against ISO-induced oxidative stress by augmenting the antioxidant defense system and mitigating oxidative damage. This protective effect highlights the therapeutic potential of CVC in addressing disorders related to oxidative stress.

### CVC’s neuroprotective effects: reduction of ISO-induced brain injury markers and inflammatory responses

**Fig. 2:**
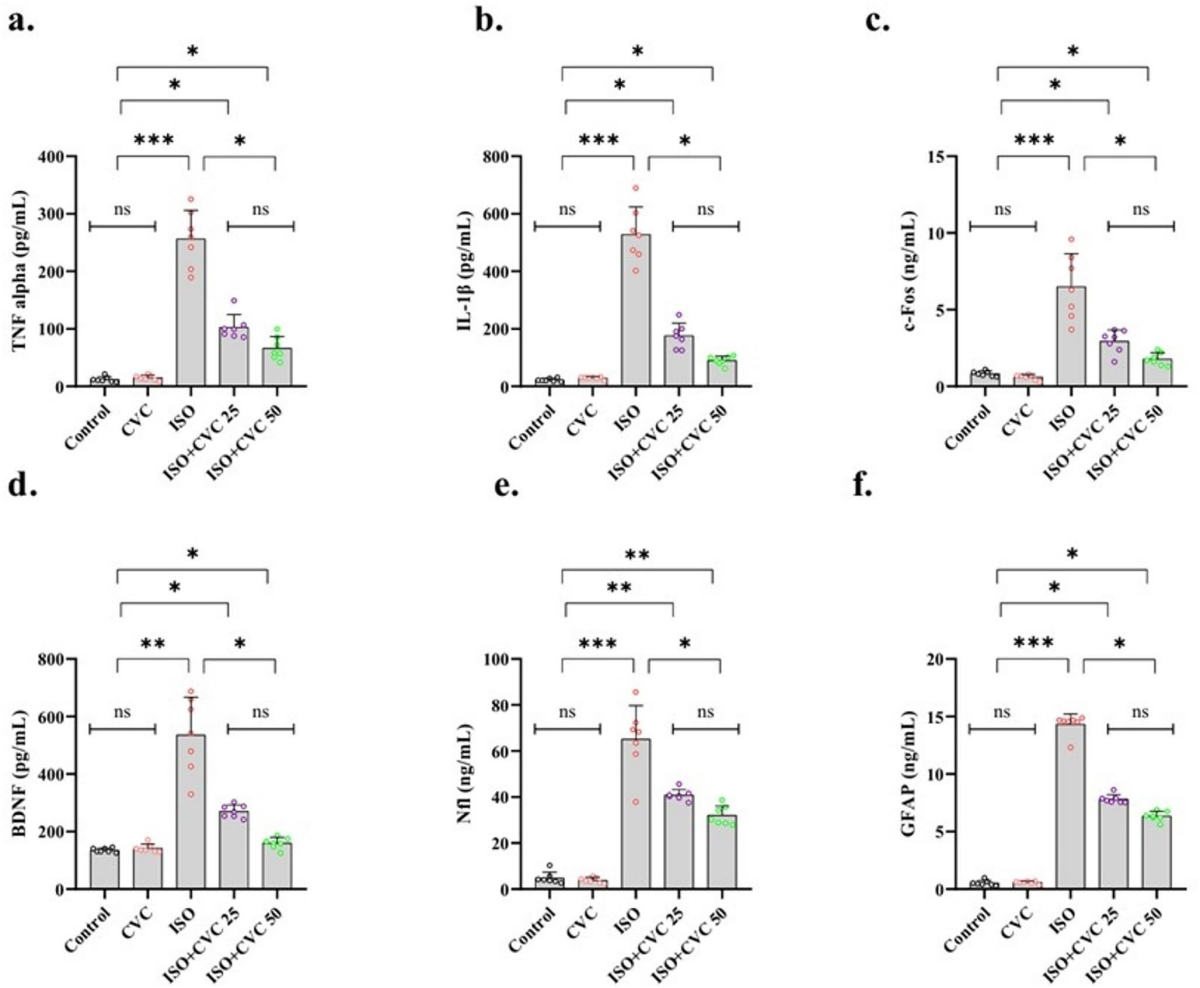
Effects of Carvacrol (CVC) on Brain Damage and Neuroinflammatory Markers in ISO-Induced Rats. (**a**-**f**) Expression levels of TNF-alpha, IL-1β, c-Fos, BDNF, Nfl, and GFP were evaluated in the experimental groups. The control and CVC-only groups showed no significant differences in the expression of these markers, indicating that CVC alone does not alter baseline levels (*p* > 0.05). The ISO-induced group exhibited significantly elevated expression of all markers compared to the control (*p* < 0.0001), reflecting robust activation of inflammatory pathways, neuronal and glial damage, and injury responses. In the CVC-treated group post-ISO exposure, a significant reduction in the expression of these markers was observed (*p* < 0.01). Specifically, TNF-alpha and IL-1β reductions reflect decreased neuroinflammation. BDNF and c-Fos modulations suggest normalized neuronal activity and plasticity. Decreased Nfl and GFP indicate reduced neuronal and glial damage. These results underscore CVC’s neuroprotective properties in mitigating ISO-induced brain injury and inflammatory stress. Data are presented as mean ± SD, with statistical significance denoted as *p* < 0.05, *p* < 0.01, and *p* < 0.0001

The analysis of brain damage markers indicated no substantial alterations in the expression levels of TNF-alpha, IL-1β, c-Fos, BDNF, Nfl, and GFP between the control group and the CVC-only therapy group (*p* > 0.05). The results indicate that CVC injection, without causing injury, does not modify the baseline expression of these markers, preserving a steady-state condition (Fig. 2a-f).

A considerable elevation in the expression levels of all assessed markers—TNF-alpha, IL-1β, c-Fos, BDNF, Nfl, and GFP—was noted in the ISO-induced group, exhibiting extremely significant differences relative to the control group (*p* < 0.0001). This discovery signifies a strong activation of inflammatory and injury-related pathways, along with neuronal and glial responses, in ISO-induced circumstances (Fig. 2a-f).

Significantly, in the cohort administered CVC post-ISO induction, the expression levels of these markers were markedly diminished relative to the ISO group (*p* < 0.01). This reduction underscores the capacity of CVC to alleviate the inflammatory response (evidenced by diminished TNF-alpha and IL-1β expression) and to modulate neuronal activity and plasticity (as demonstrated by alterations in c-Fos and BDNF levels). The reduction in Nfl and GFP expression indicates a protective function of CVC in diminishing indicators of neuronal and glial damage, hence corroborating its neuroprotective characteristics (Fig. 2a-f).

These data indicate that CVC does not disrupt normal physiological marker expression, yet it has a substantial protective effect against ISO-induced brain injury. This indicates that CVC may have a therapeutic function in mitigating neuroinflammation and neuronal injury in circumstances marked by high oxidative or inflammatory stress.

### No significant changes in Abeta40, pTau181, and tTau expression across all experimental groups

**Fig. 3:**
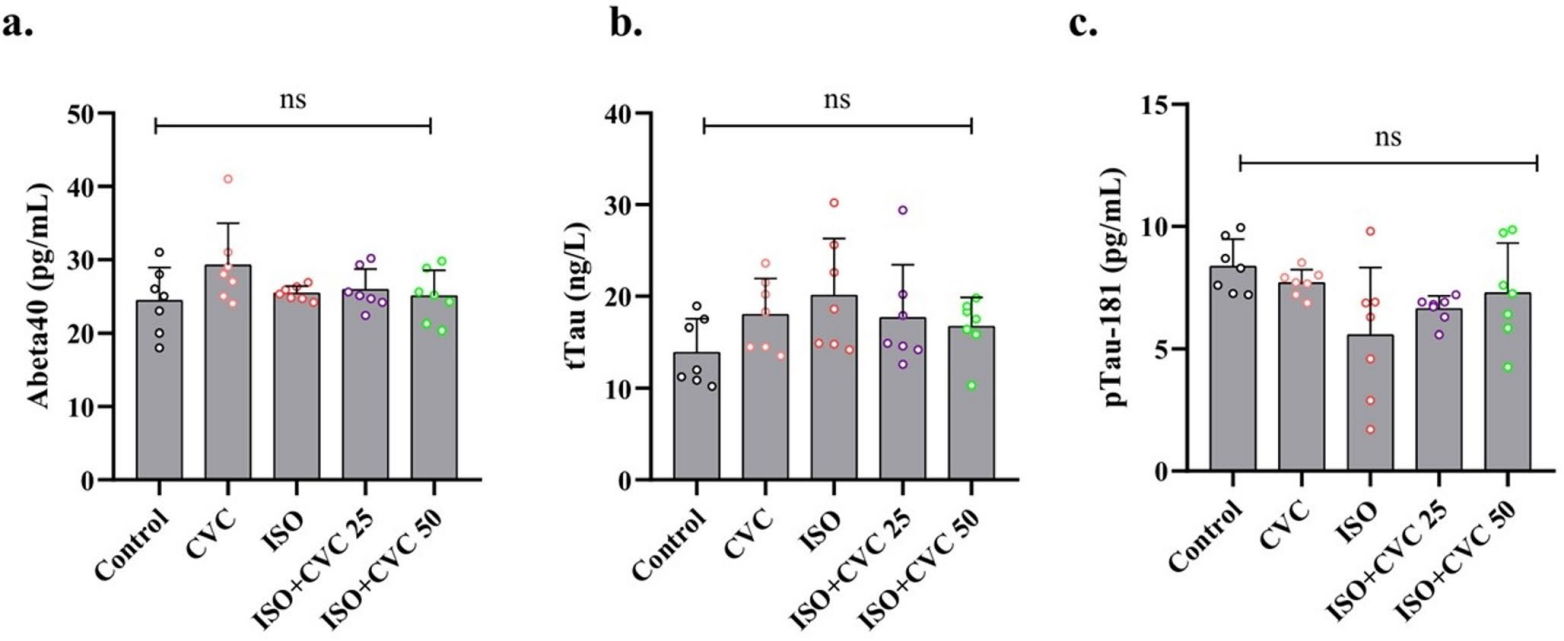
Expression Levels of Neurodegenerative Biomarkers (Abeta40, pTau181, and tTau) Across Experimental Groups. (**a**-**c**) Expression levels of Abeta40, pTau181, and tTau, biomarkers commonly associated with neurodegenerative conditions such as Alzheimer’s disease, were analyzed across all experimental groups. No statistically significant differences were observed between the control, ISO-induced, CVC-only, and ISO + CVC groups (*p* > 0.05), indicating that these biomarkers remained stable under the study’s experimental conditions and timeframe. These results suggest that the treatments and interventions, including ISO administration and CVC therapy, did not notably affect the expression levels of these neurodegenerative markers. The stability of these biomarkers implies that the observed protective effects of CVC on oxidative stress, neuroinflammation, and mitochondrial dysfunction were not directly linked to changes in Abeta40, pTau181, or tTau levels. Data are presented as mean ± SD, with no significant changes detected across groups

No statistically significant changes were seen in the expression levels of Abeta40, pTau181, and tTau across all experimental groups (*p* > 0.05). This suggests that, within the parameters of this study, the concentrations of these biomarkers, typically linked to neurodegenerative disorders like Alzheimer’s disease, remained comparatively steady. Notwithstanding possible changes in other biochemical markers, the expression of Abeta40, pTau181, and tTau showed no significant variations in response to the administered treatments or interventions, indicating that these particular markers may remain largely unaffected under the experimental conditions or timeframe employed (Fig. 3a-c). Consequently, these findings suggest that, at least within the confines of this investigation, the observed alterations in other parameters were not correlated with variations in the levels of these essential neurodegenerative indicators.

### CVC attenuates ISO-induced mitochondrial damage: a reduction in mRNA transcript levels of NSE, s100B, CALP1, and CALM1

**Fig. 4:**
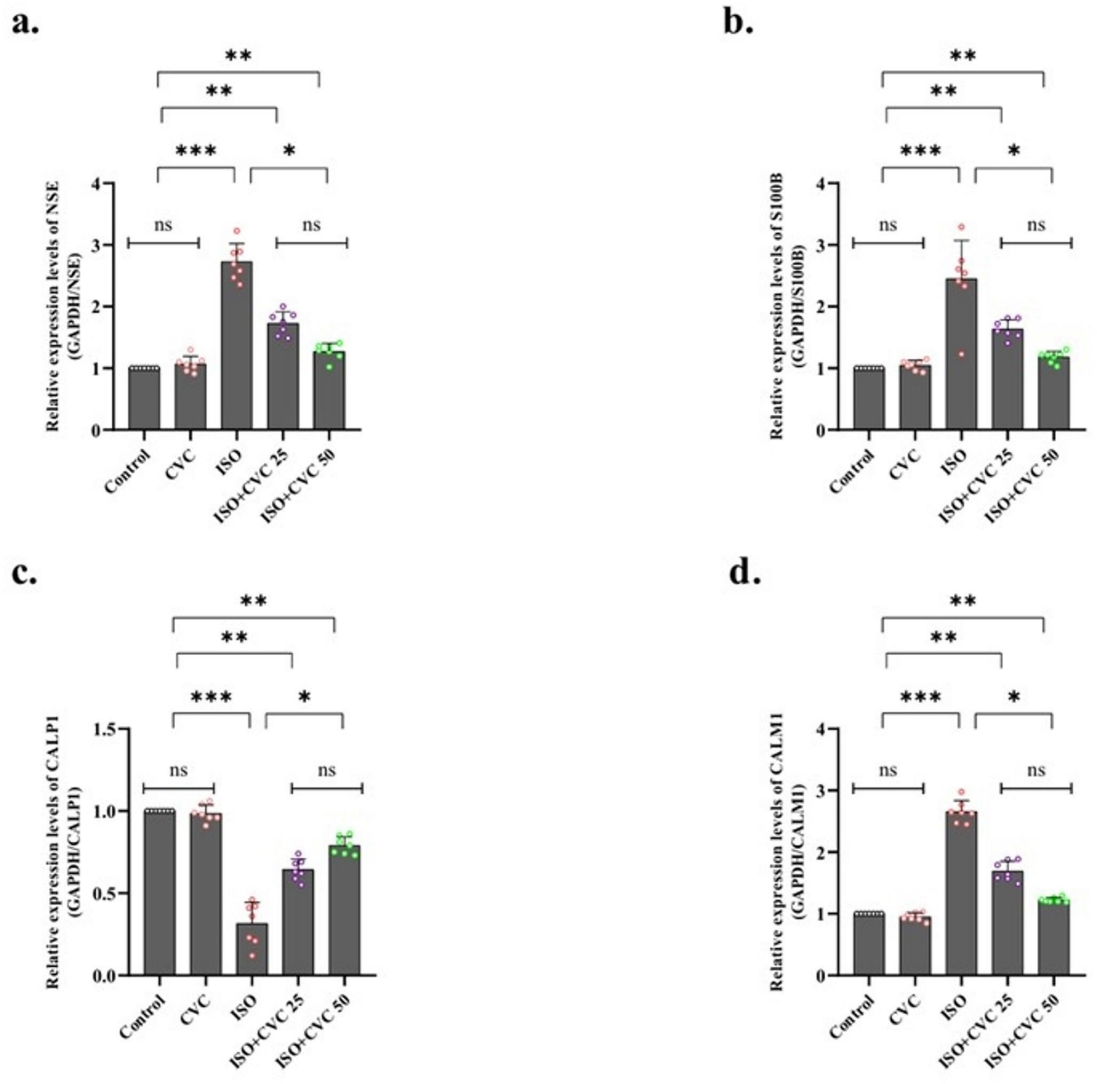
Effects of Carvacrol (CVC) on Mitochondrial Damage Markers in ISO-Induced Rats. (**a**-**d**) mRNA transcript levels of mitochondrial damage markers, including NSE, s100B, CALP1, and CALM1, were analyzed across experimental groups. No significant changes were observed between the control and CVC-only groups (*p* > 0.05), suggesting that CVC administration does not alter baseline mitochondrial integrity under normal conditions. A significant upregulation of these markers was detected in the ISO-induced group compared to the control group (*p* < 0.0001), indicating substantial mitochondrial dysfunction and cellular stress caused by ISO administration. In the ISO + CVC-treated group, the expression levels of these markers were significantly reduced compared to the ISO group (*p* < 0.01), indicating that CVC mitigated ISO-induced mitochondrial damage and helped restore mitochondrial integrity. These results highlight CVC’s protective effects against ISO-induced mitochondrial dysfunction while showing no adverse effects under normal conditions. Data are presented as mean ± SD, with statistical significance denoted as *p* < 0.05, *p* < 0.01, and *p* < 0.0001

Analysis of mitochondrial damage indicators revealed that the mRNA transcript levels of NSE, s100B, CALP1, and CALM1 were consistent in both the control and CVC treatment groups (*p* > 0.05). The data indicate that, under standard conditions and after CVC therapy, there is no substantial change in the expression of mitochondrial damage markers, suggesting that CVC does not adversely affect mitochondrial integrity under current circumstances (Fig. 4a-d).

A notable elevation in the mRNA transcript levels of the markers—NSE, s100B, CALP1, and CALM1—was detected in the ISO-induced group (*p* < 0.0001). This rise indicates a significant upregulation due to ISO-induced mitochondrial dysfunction, aligning with the established effects of ISO in inducing cellular stress and damage. The increased mRNA levels of these markers indicate the activation of mitochondrial damage pathways, potentially contributing to neurodegenerative diseases (Fig. 4a-d).

In the CVC application group, there was a significant decrease in the mRNA transcript levels of these markers (*p* < 0.01) following ISO-induced mitochondrial stress. This reduction suggests that CVC therapy may confer a protective impact on mitochondrial integrity, presumably by alleviating the oxidative and cellular stress caused by ISO. The restoration of these marker levels indicates that CVC may significantly contribute to mitigating mitochondrial damage and maintaining cellular function following induced injury (Fig. 4a-d).

These findings combined indicate that although CVC does not modify mitochondrial damage markers under normal circumstances, it significantly mitigates the elevation of these markers induced by ISO-related mitochondrial dysfunction. This indicates that CVC may provide therapeutic potential in safeguarding against mitochondrial damage and associated cellular processes.

**Fig. 5:**
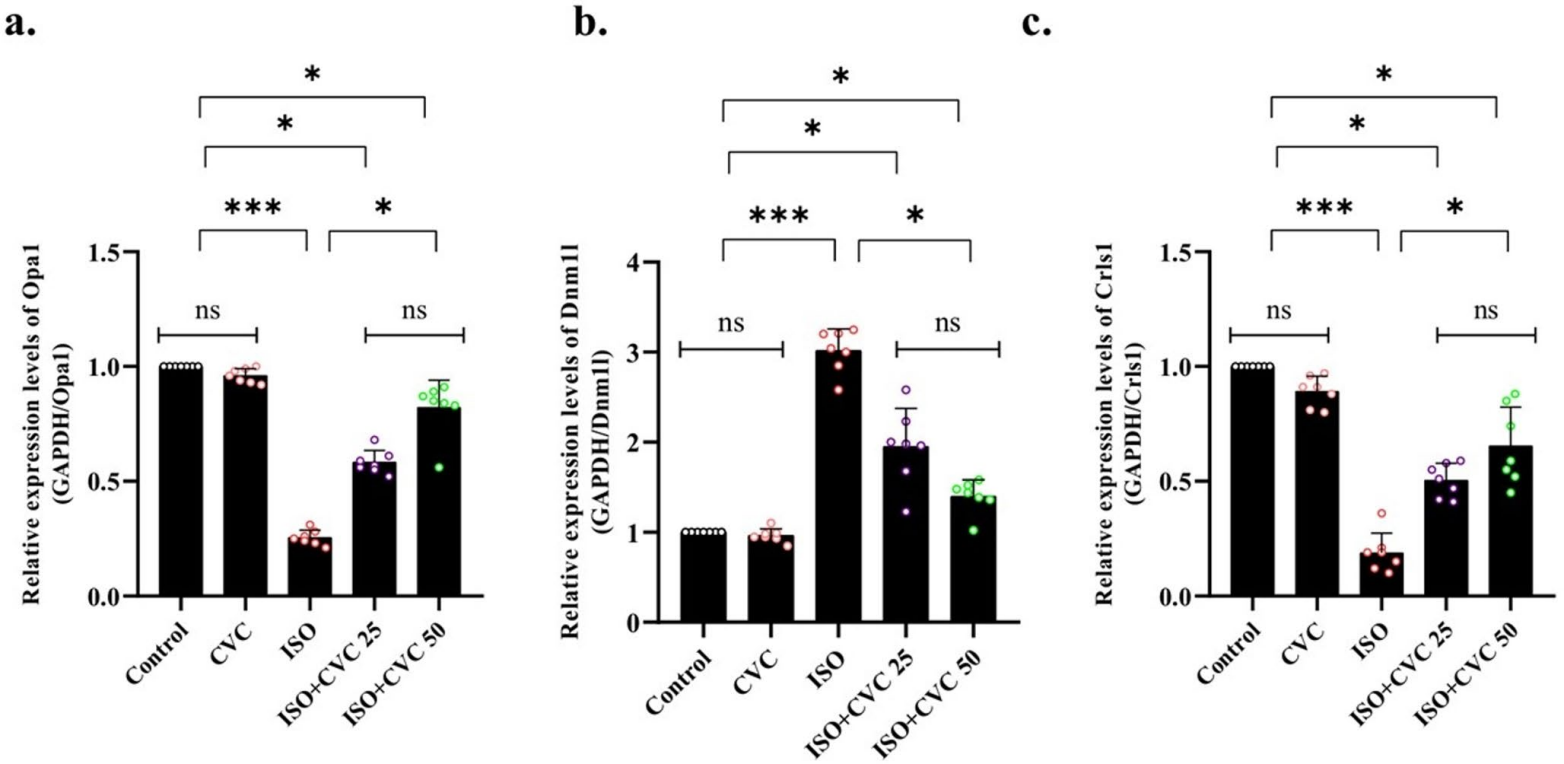
Effects of Carvacrol (CVC) on Mitochondrial Damage Markers in ISO-Induced Rats. (**a**-**c**) mRNA transcript levels of mitochondrial damage markers, including Opa1, DNM1I, and Crls1 were analyzed across experimental groups

In the ISO group, the mRNA transcript levels of Opa1 and Crls1 were downregulated, while CVC treatment elevated their expression. This indicates that CVC may provide neuroprotection by maintaining mitochondrial dynamics and functionality (Fig. 5a and c). In the ISO group, Dnm1 l mRNA transcript levels were elevated, but CVC treatment resulted in a substantial decrease. This suggests that CVC may assist in regulating mitochondrial dynamics by mitigating the excessive fission linked to ISO-induced stress (Fig. 5b).

### CVC modulates IL-1β and β-Amyloid levels in ISO-treated rats

**Fig. 6:**
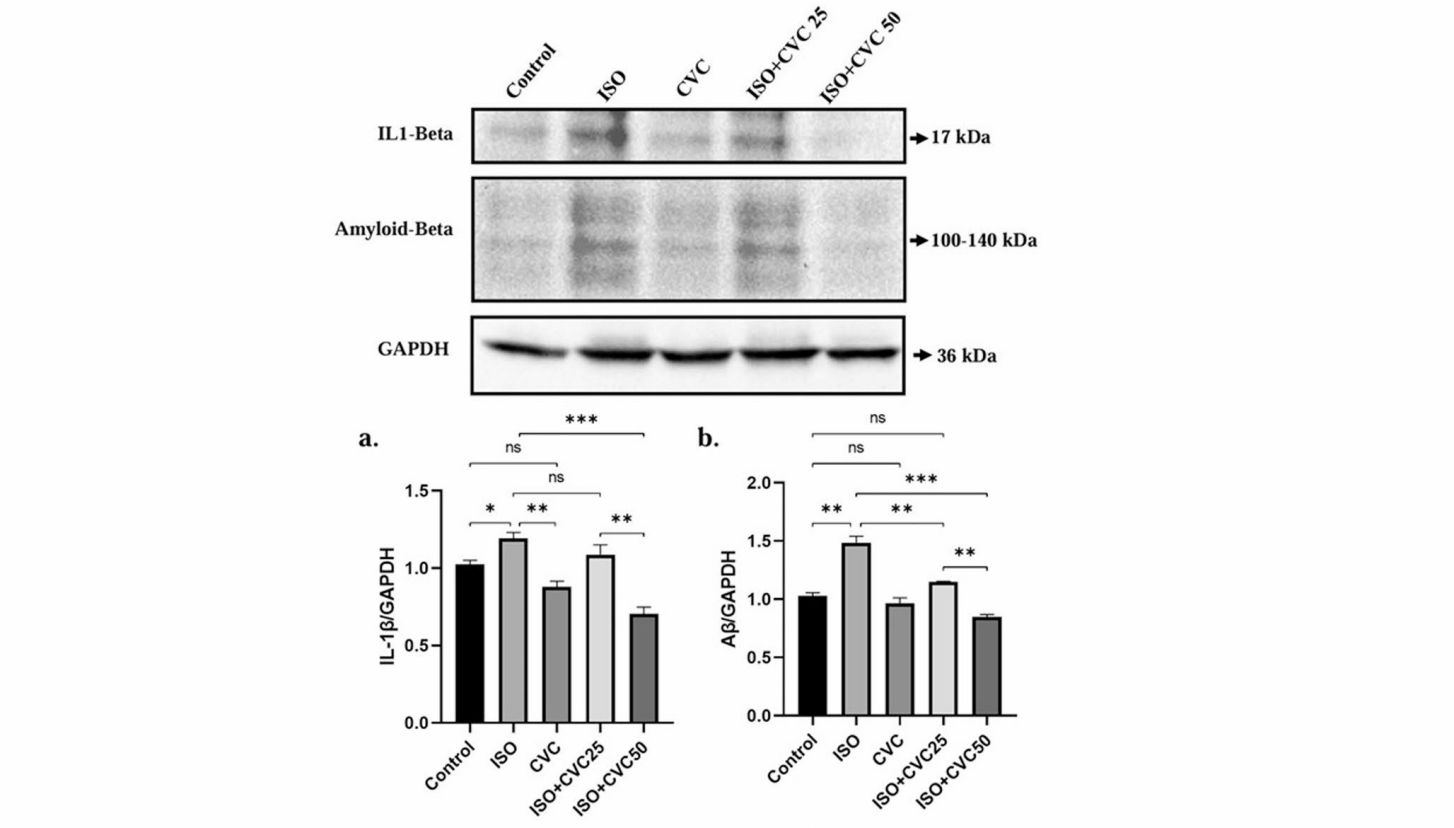
Effects of CVC on IL-1β and β-Amyloid Levels in ISO-Induced Rats. Western blot (WB) analysis was performed to assess the expression levels of IL-1β and β-amyloid across the experimental groups. IL-1β expression was significantly elevated in the ISO group compared to the control (*p* < 0.05), reflecting a robust inflammatory response triggered by ISO-induced oxidative stress and neuroinflammation. In the CVC50 therapy group, IL-1β levels were significantly reduced (*p* < 0.0001), indicating that CVC50 has anti-inflammatory effects and may attenuate the ISO-induced inflammatory response, restoring a more balanced cytokine profile. β-amyloid levels were significantly increased in the ISO group (*p* < 0.01), suggesting that ISO may contribute to amyloid formation, a hallmark of neurodegenerative diseases. On the other hand, while CVC treatment at both doses reduced amyloid formation, CVC50 proved to be more effective. Data are presented as mean ± SD, with statistical significance indicated as *p* < 0.05, *p* < 0.01, and *p* < 0.0001

Western blot analysis was conducted to assess the protein levels of IL-1β and β-amyloid in the experimental groups. The findings demonstrated that IL-1β levels were significantly increased in the ISO group relative to the control (*p* < 0.01), signifying a pronounced inflammatory response triggered by ISO treatment. The elevation of IL-1β indicates an intensified pro-inflammatory condition, typically linked to oxidative stress and neuroinflammation (Fig. 6a). In the ISO + CVC50 group, a notable decrease in IL-1β levels was observed compared to the ISO group (*p* < 0.0001), suggesting that CVC50 may attenuate the ISO-induced inflammatory response. This reduction indicates that CVC may possess anti-inflammatory capabilities, aiding in the restoration of equilibrium by diminishing the excessive production of pro-inflammatory cytokines such as IL-1β after ISO-induced stress (Fig. 6a).

Examination of β-amyloid levels revealed a significant elevation in the ISO group compared to the control group (*p* < 0.001), corroborating the idea that ISO treatment may induce heightened amyloid formation, a characteristic of neurodegenerative diseases (Fig. 6b). However, the ISO + CVC25 (*p* < 0.001) and ISO + CVC 50 (*p* < 0.0001) groups showed significantly decreased levels of β-amyloid compared to the ISO group.

### Protective effects of CVC against ISO-induced histopathological changes in the cerebral cortex and hippocampal CA3 region

**Fig. 7:**
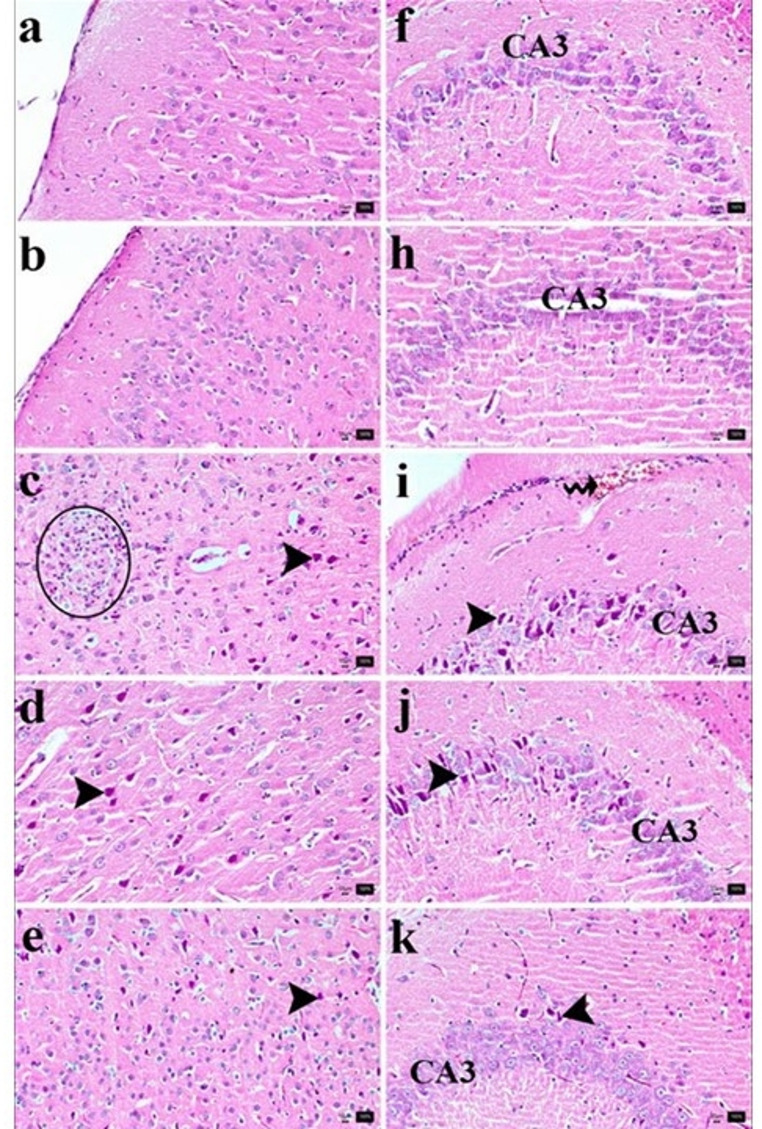
Histological Analysis of Neuronal Damage in the Cerebral Cortex and Hippampal CA3 Region of ISO-Induced Rats Treated with Carvacrol. Histological examination of the cerebral cortex and hippocampal CA3 region was conducted to assess neuronal integrity and damage across experimental groups. These histological findings indicate that CVC50 significantly ameliorates neuronal damage in both the cerebral cortex and hippocampal CA3 region following ISO-induced injury. Data are presented as mean ± SD, with statistical significance denoted as *p* < 0.05

The lesion scores for the cerebral cortex and hippocampal CA3 regions of the different experimental groups are summarized in Table 1. Microscopic examination of cerebral cortex sections from the control and CVC50 groups revealed normal brain histology, including intact neurons and glial cells (Fig. 7a and b). Neurons displayed centrally located vesicular nuclei, consistent with healthy morphology. In contrast, the cerebral cortex of rats in the isoproterenol (ISO) group exhibited significant pathological changes, including eosinophilic, shrunken, and angular necrotic neurons, accompanied by gliosis. Furthermore, neuronophagia, characterized by microglial cells surrounding necrotic neurons, was observed (Fig. 7c; Table 2).

**Table 1:** Primer squences and informations

	Primers	Product Size (bp)	Accesion Number
NSE	F: GGGCACTCTACCAGGACTTT R: CAAACAGTTGCAGGCCTTCT	191	AF019973.1
S100B	F: CAGGGAGAGAGGGTGACAAG R: AACTCATGACAGGCTGTGGT	208	BC087026.1
CALP1	F: ATCCCACCTGCAGTCATCTC R: CCCATCATCTCTCTGCCCAT	205	XM_032905553.1
CALM1	F: TGATAAAGATGGGGACGGCA R: GCCATTGCCATCCTTGTCAA	238	AF178845.1
Opa1	F: CTGGACAAGATCGCCGAAAG R: TTCTCCAAACGCTCCAGGAT	155	NM_001433910.1
Dnm1 l	F: GCAACTGGAGAGGAATGCTG R: CACAATCTCGCTGTTCTCGG	174	NM_053655.3
Crls1	F: CACCGCGAACACTAGCTAAG R: GGACGCAGCTGTAGTGAATG	197	NM_001014258.1
GAPDH	F: AACGACCCCTTCATTGACCT R: CCCCATTTGATGTTAGCGGG	164	NM_017008.4

Neuron-Specific Enolase (NSE), S100 calcium binding protein B (S100B), Calbindin (CALB1), Calmodulin (CALM1), mitochondrial dynamin like GTPase (Opa1), dynamin 1-like (Dnm1 l), cardiolipin synthase 1 (Crls1), glyceraldehyde-3-phosphate dehydrogenase (GAPDH)

**Table 2:** Statistical scores of histopathological findings in the cerebral cortex and hippocampal CA3

Groups	Mean ± Standard error
Control/Brain Cortex	0.14 ± 0.14^a^
CVC50/Brain Cortex	0.00 ± 0.00^a^
İZO/Brain Cortex	2.85 ± 0.14^b^
İZO + CVC 25/Brain Cortex	2.42 ± 0.29^b^
İZO + CVC 50/Brain Cortex	1.00 ± 0.21^c^
Control/CA3	0.00 ± 0.00^a^
CVC 50/CA3	0.00 ± 0.00^a^
İZO/CA3	2.71 ± 0.18^b^
İZO + CVC 25/CA3	2.14 ± 0.34^b^
İZO + CVC 50/CA3	0.71 ± 0.18^c^

In the ISO + CVC25 group, neuronal damage persisted, manifesting as degeneration and necrosis, though the severity was somewhat attenuated (Fig. 7d). However, in the ISO + CVC50 group, neuronal damage in the cerebral cortex was markedly reduced, and a statistically significant improvement was observed compared to the ISO and ISO + CVC25 groups (Fig. 7e, *p* < 0.05, Table 2).

In the hippocampal CA3 region, a large number of Nissl bodies were evident in the cytoplasm of neurons within the polymorphic cell layer in the control and CVC50 groups (Fig. 7f and h). Conversely, the hippocampal CA3 region of rats treated with ISO showed pathological congestion, shrunken hyperchromatic pyknotic nuclei, and cells with eosinophilic cytoplasm (Fig. 7i). Similarly, in the ISO + CVC50 group, necrotic pyramidal cells with pyknotic and eosinophilic characteristics, resembling those in the ISO group, were still prominent (Fig. 7j; Table 2).

In contrast, the ISO + CVC50 group demonstrated a significant reduction in neuronal necrosis in the hippocampal CA3 region, with a statistically significant difference noted when compared to both the ISO and ISO + CVC25 groups (Fig. 7k, *p* < 0.05, Table 2).

## Discussion

This study sought to investigate the possible therapeutic effects of CVC on oxidative stress, neuroinflammation, and mitochondrial dysfunction in a rat model produced by ISO. The findings offer significant insights into the preventive function of CVC against the detrimental effects of oxidative stress and cerebral damage. The results indicate that CVC significantly influences oxidative stress markers, neuroinflammatory pathways, mitochondrial damage, and particular brain injury markers, hence reinforcing its potential as a neuroprotective drug.

Oxidative stress significantly contributes to cellular damage and is crucial in numerous neurological illnesses (Pizzino et al. 2017; Salim 2017; Dash et al. 2024). This study’s investigation of oxidative stress markers revealed that CVC administration did not significantly affect the baseline levels of antioxidant enzymes, including SOD, CAT, GSH, and GPx, in both the control and CVC-treated groups. This indicates that CVC, when given independently, does not disrupt normal antioxidant defense mechanisms in physiological settings. Nonetheless, under ISO-induced oxidative stress, a notable decrease in these antioxidant enzymes was recorded, alongside a substantial rise in MDA, a marker of lipid peroxidation. These findings align with prior research that emphasizes ISO’s capacity to provoke oxidative stress, resulting in a compromised antioxidant defense mechanism and ensuing cellular damage (Sharma et al. 2023).

Notably, in the CVC-treated groups after ISO delivery, the levels of SOD, CAT, GSH, and GPx were dramatically restored, indicating that CVC possesses a potent antioxidative action, presumably by augmenting the antioxidant defense system. Moreover, the noted reduction in MDA levels offers conclusive proof that CVC alleviates lipid peroxidation and oxidative damage. The findings highlight the therapeutic efficacy of CVC in mitigating oxidative damage, a characteristic of numerous neurodegenerative disorders. The restoration of antioxidant enzyme levels and the lowering of MDA levels indicate that CVC may be efficacious in mitigating oxidative stress-related disorders by enhancing cellular resilience and preventing additional damage.

Neuroinflammation is a significant factor in neurological injury, especially as a reaction to oxidative stress (Adegbola et al. 2017; Giri et al. 2024; Suleiman Khoury et al. 2024; Mao et al. 2024). Our investigation demonstrated that ISO-induced damage resulted in a substantial increase in the expression levels of critical inflammatory and injury-related markers, including TNF-alpha, IL-1β, c-Fos, BDNF, Nfl, and GFP. These markers are acknowledged as biomarkers of neuroinflammation, neuronal activity, and damage (Culjak et al. 2021; Zhang et al. 2024), with their increase signifying a robust inflammatory and injury response subsequent to ISO treatment. Elevated levels of TNF-alpha and IL-1β indicate a significant pro-inflammatory condition, although changes in c-Fos and BDNF signify the activation of neural plasticity and activity in response to injury.

CVC injection after ISO exposure led to a notable decrease in the expression of these markers. The decrease in TNF-alpha and IL-1β indicates that CVC successfully mitigates the inflammatory response triggered by ISO. The downregulation of c-Fos and BDNF suggests that CVC may influence neuronal plasticity and activity, thereby reducing neuronal damage and facilitating recovery. Moreover, the reduced expression of Nfl and GFP, indicators of neuronal and glial damage, reinforces the neuroprotective properties of CVC. These results align with other research demonstrating the importance of neuroinflammation in the advancement of neurodegenerative disorders, and the outcomes of this study underscore CVC’s potential in mitigating neuroinflammatory responses and safeguarding against neuronal damage (Zamanian et al. 2021; Azizi et al. 2022).

Mitochondrial malfunction is pivotal to several types of cellular damage and is significantly associated with neurodegenerative disorders (Hroudová et al. 2014; Norat et al. 2020; Alqahtani et al. 2023; Zong et al. 2024). This work evaluated the mRNA expression levels of mitochondrial damage indicators (NSE, s100B, CALP1, and CALM1) to evaluate the effects of ISO-induced mitochondrial stress. As anticipated, ISO therapy resulted in a substantial elevation of mRNA levels for these markers, indicating that ISO promotes mitochondrial dysfunction and activates cellular damage pathways. This augmentation aligns with prior research emphasizing ISO’s adverse impact on cellular integrity, particularly concerning mitochondrial impairment (Naaz et al. 2020; Qian et al. 2024).

CVC treatment following ISO exposure led to a notable decrease in the expression levels of mitochondrial damage markers. This reduction suggests that CVC may confer a protective impact on mitochondrial function, presumably by mitigating the oxidative and cellular stress caused by ISO. The restoration of mitochondrial damage markers indicates that CVC may aid in maintaining mitochondrial integrity, potentially mitigating the advancement of mitochondrial dysfunction and associated cellular damage. These findings are significant, indicating that CVC may contribute to mitochondrial protection, a domain that requires more exploration due to its relevance in numerous neurodegenerative disorders.

β-amyloid accumulation plays a pivotal role in the pathogenesis of neurodegenerative disorders, including Alzheimer’s disease (Solis et al. 2020; Celik Topkara et al. 2022; Caputo et al. 2023; Tareen et al. 2024). In this study, β-amyloid levels were significantly elevated in the ISO group, suggesting that ISO exposure may promote amyloid buildup. CVC treatment led to a marked reduction in β-amyloid levels, indicating its potential role in modulating amyloid metabolism. The observed decrease in β-amyloid accumulation suggests that CVC may counteract ISO-induced disruptions, thereby alleviating the amyloid burden. This finding points to a potential neuroprotective mechanism of CVC, which may involve regulating β-amyloid production, enhancing clearance, or preventing aggregation. However, given the complex interplay of amyloid metabolism, deposition, and clearance pathways, further studies are needed to elucidate the precise mechanisms through which CVC exerts its effects.

This study evaluated the impact of CVC on specific markers often linked to neurodegenerative disorders, including Abeta40, pTau181, and tTau. These indicators are frequently raised in illnesses such as Alzheimer’s disease and are routinely employed to evaluate neuronal damage and tauopathies (Delaby et al. 2022; Park et al. 2024). Nonetheless, this analysis revealed no statistically significant alterations in the expression levels of Abeta40, pTau181, and tTau across all experimental groups. This indicates that, within the experimental parameters and duration of the investigation, the concentrations of these particular biomarkers were not substantially influenced by ISO therapy or CVC administration. Considering that ISO is known to trigger various clinical processes, additional studies with prolonged exposure or varying experimental conditions are needed to elucidate the exact mechanisms underlying the absence of notable alterations in these markers.

This study’s findings illustrate the protective benefits of CVC, especially at elevated doses, against ISO-induced histopathological damage in the cerebral cortex and hippocampal CA3 region (Zare Mehrjerdi et al. 2020). In the ISO group, considerable neuronal injury was noted, including necrosis, gliosis, and neuronophagia in the cerebral cortex, with congestion and necrotic alterations in the hippocampal CA3 area. These results align with the established neurotoxic effects of ISO, which provokes oxidative stress and inflammation, resulting in cellular damage (Khan et al. 2024). CVC, a phenolic monoterpene known for its established antioxidant and anti-inflammatory effects, demonstrated the ability to alleviate these pathogenic alterations in a dose-dependent fashion. The ISO + CVC25 group had only moderate enhancement, but the ISO + CVC50 group displayed markedly decreased neuronal necrosis and enhanced histological integrity in both the cerebral cortex and hippocampal CA3 region. The decrease in neuronal damage indicates that CVC may have protective effects by mitigating ISO-induced oxidative stress and inflammation, as evidenced by prior research emphasizing its neuroprotective capabilities (Javed et al. 2023).

The maintenance of Nissl bodies in hippocampal neurons and the decreased extent of eosinophilic necrosis in the cerebral cortex further highlight the effectiveness of CVC at elevated doses (Amooheydari et al. 2022). These findings correspond with previous studies highlighting CVC’s significance in maintaining neuronal integrity and functionality under stress situations.

The notable enhancement seen in the ISO + CVC50 group underscores the therapeutic efficacy of CVC in alleviating neurodegenerative mechanisms linked to oxidative and inflammatory injury. Additional research is necessary to investigate the fundamental molecular pathways and assess its enduring protective effects across various neurotoxic models.

## Conclusion

This study concludes that CVC exerts a protective effect against ISO-induced oxidative stress, neuroinflammation, and mitochondrial dysfunction. Findings suggest that CVC enhances antioxidant defenses, reduces inflammatory responses, and mitigates mitochondrial damage, potentially offering therapeutic benefits for conditions associated with oxidative and inflammatory stress. The impact of CVC on ISO-induced β-amyloid accumulation and key neurodegenerative biomarkers, such as Aβ40, pTau181, and tTau, remains unclear due to the complexity of amyloid and tau metabolism. While β-amyloid accumulation was significantly affected, these specific markers showed no notable changes, suggesting that further research is necessary to delineate CVC’s precise role in amyloid metabolism and its implications for neurodegenerative diseases. Overall, these findings underscore CVC’s potential as a neuroprotective agent and highlight the need for future studies with different time points and dosages to explore its broader therapeutic applications in neurodegenerative disorders.

## Electronic supplementary material

Below is the link to the electronic supplementary material.

Supplementary Material 1 (PNG 394 kb)

High Resolution Image (TIF 2.68 mb)

Supplementary Material 2 (PNG 731 kb)

High Resolution Image (TIF 2.68 mb)

Supplementary Material 3 (PNG 661 kb)

High Resolution Image (TIF 2.68 mb)

## Data Availability

No datasets were generated or analysed during the current study.
