# Supplementary figures and images for: Neuroprotective potential of carvacrol: restoration of oxidative balance and mitigation of brain injury markers in isoproterenol-induced rats
Source: Metab Brain Dis. 2025 May 23;40(5):211. doi: 10.1007/s11011-025-01634-6 (PMC12102131; doi:10.1007/s11011-025-01634-6)

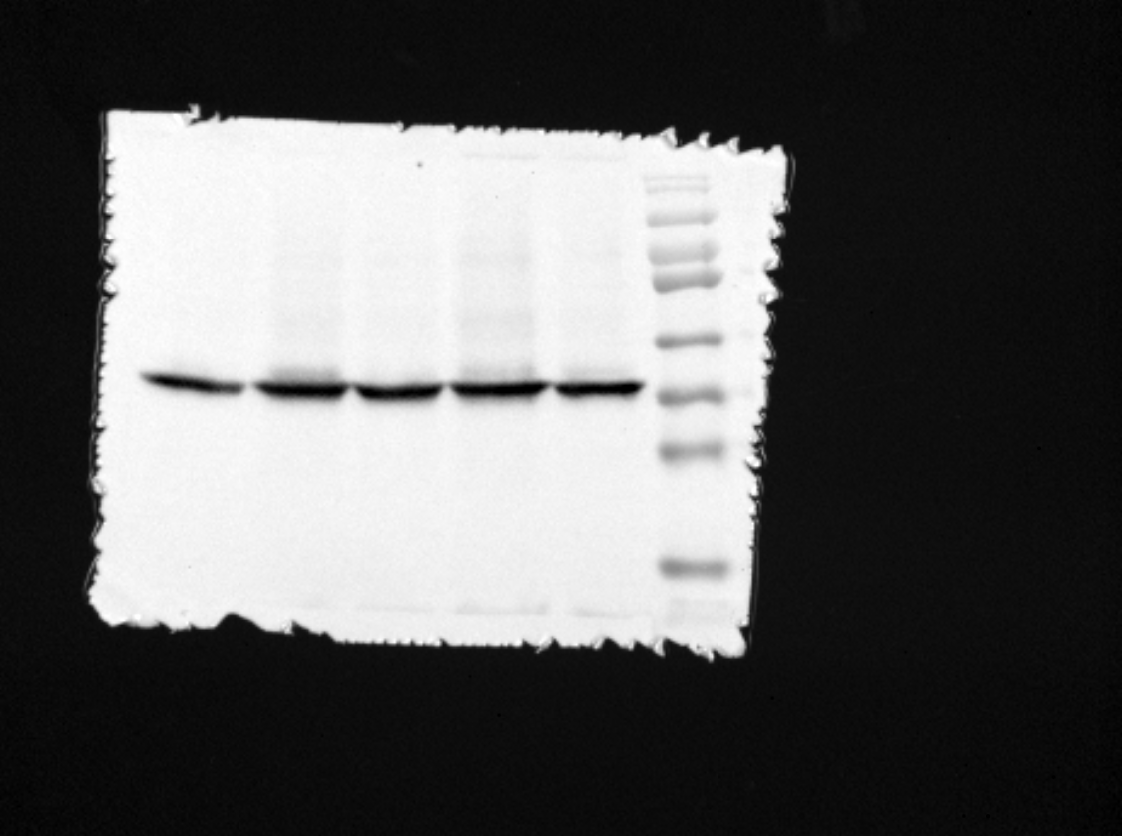

Supplement: Supplementary file 1 — Supplementary Material 1 (PNG 394 kb) [file 11011_2025_1634_Fig8_ESM.png]

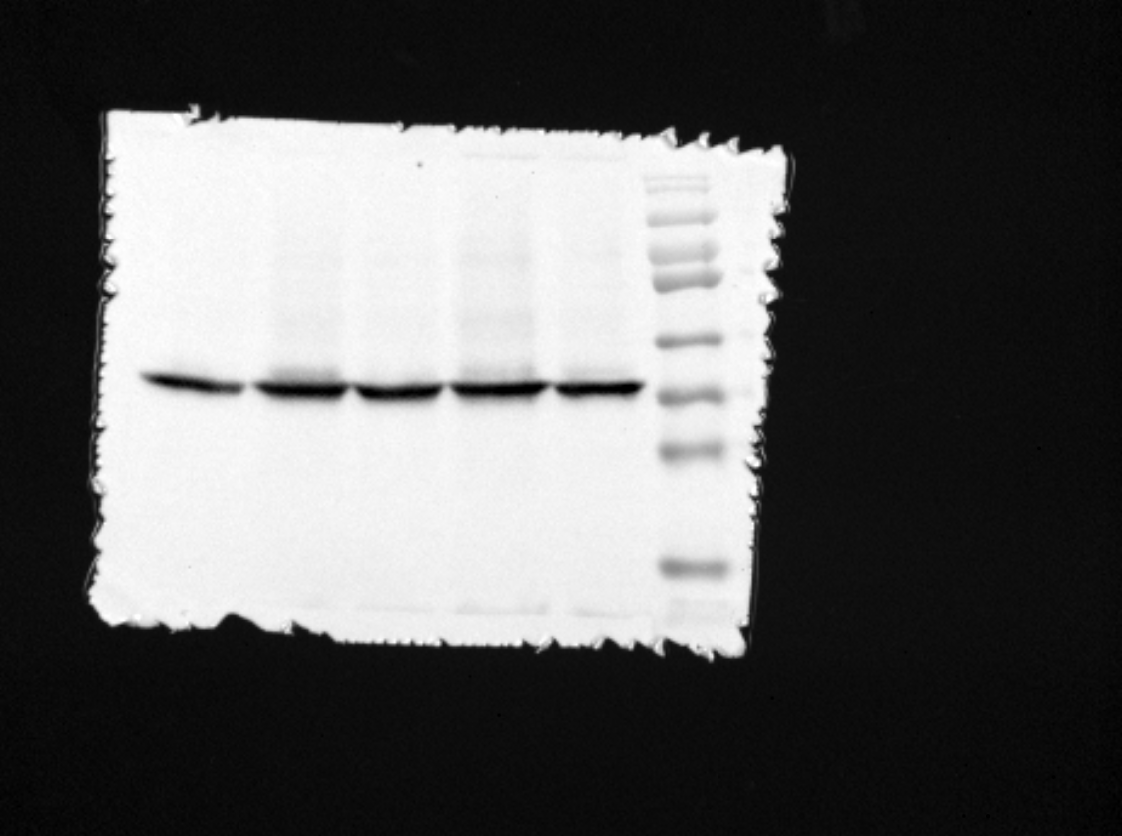

Supplement: Supplementary file 2 — High Resolution Image (TIF 2.68 mb) [file 11011_2025_1634_MOESM1_ESM.tif]

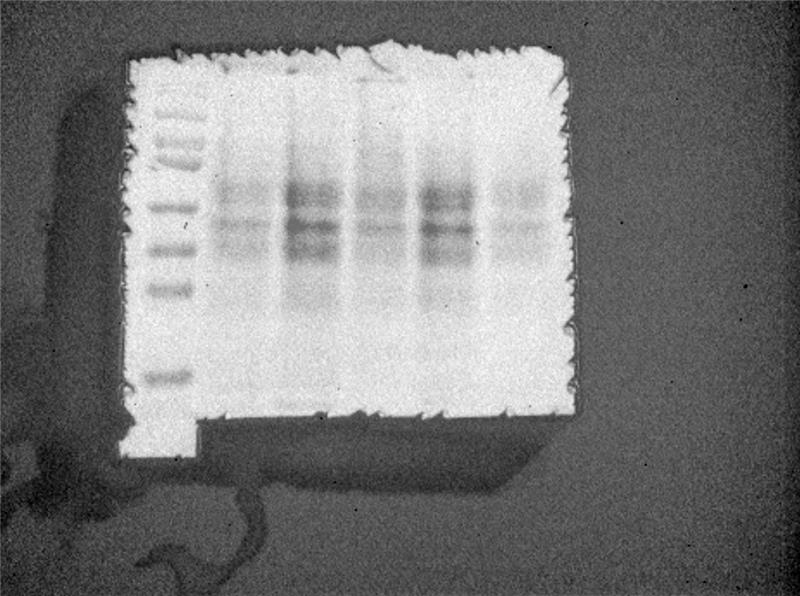

Supplement: Supplementary file 3 — Supplementary Material 2 (PNG 731 kb) [file 11011_2025_1634_Fig9_ESM.png]

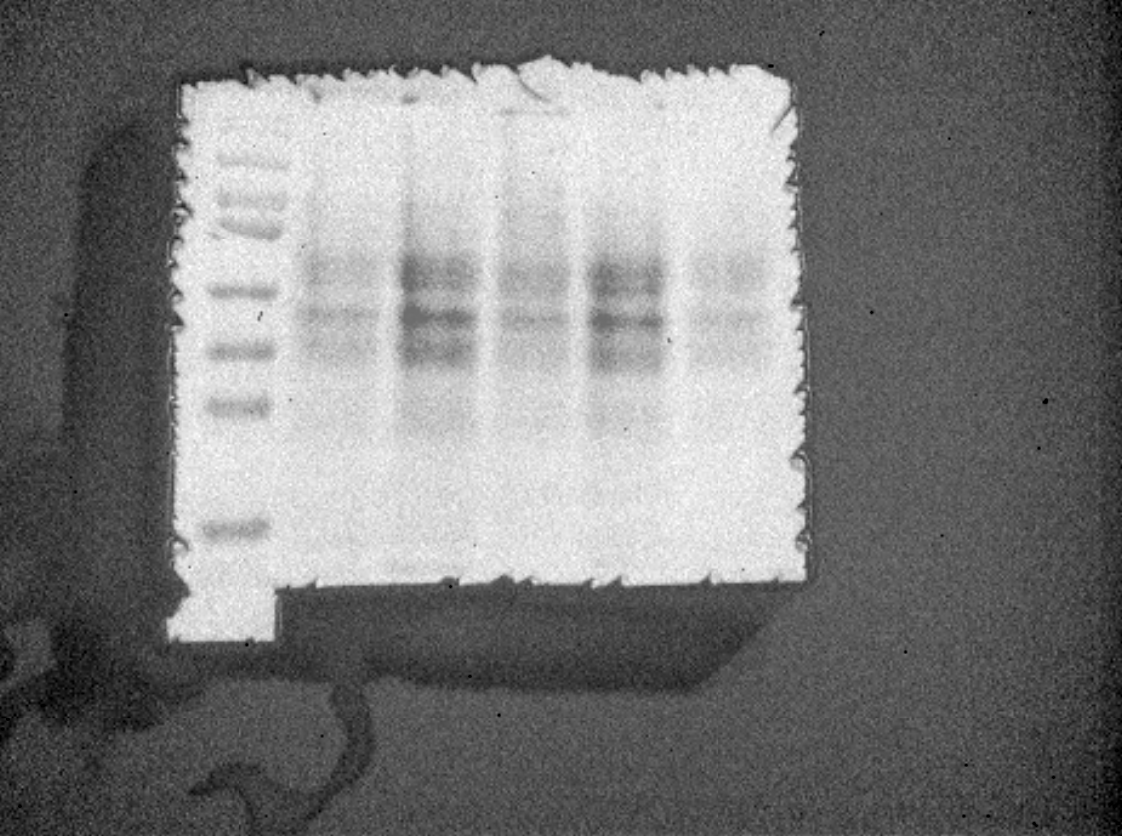

Supplement: Supplementary file 4 — High Resolution Image (TIF 2.68 mb) [file 11011_2025_1634_MOESM2_ESM.tif]

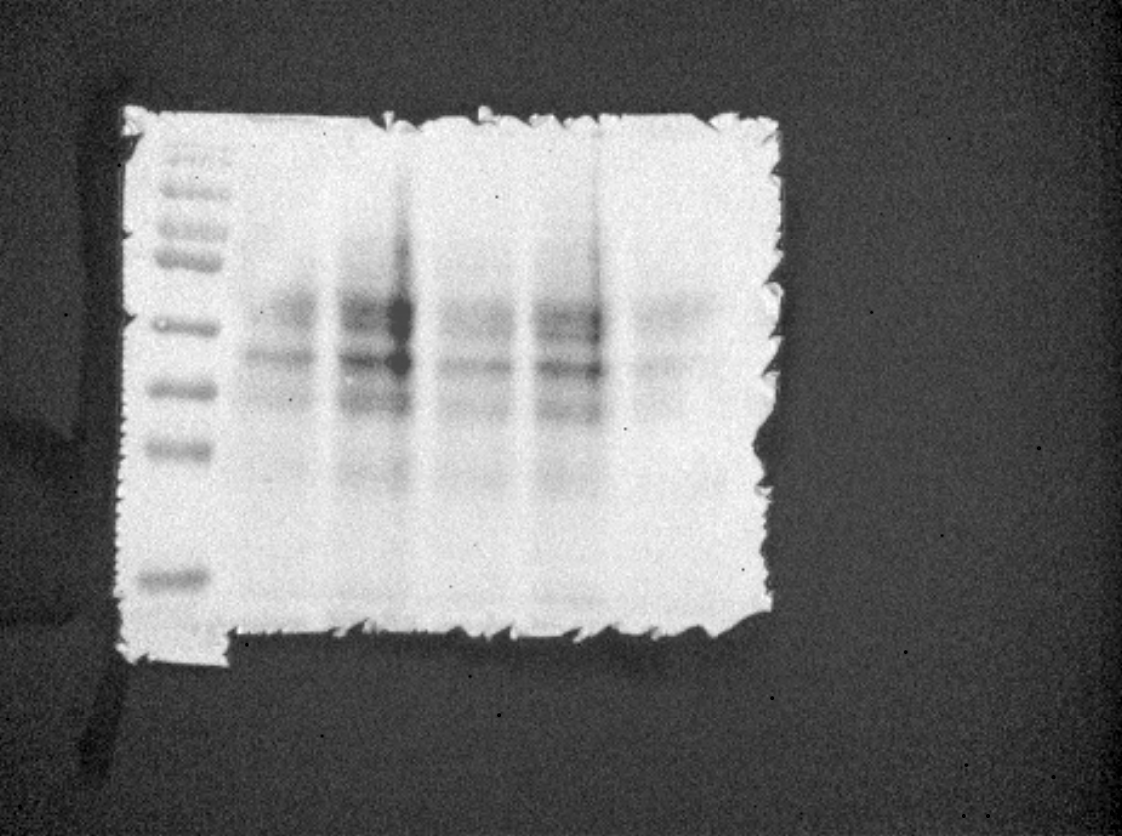

Supplement: Supplementary file 5 — Supplementary Material 3 (PNG 661 kb) [file 11011_2025_1634_Fig10_ESM.png]

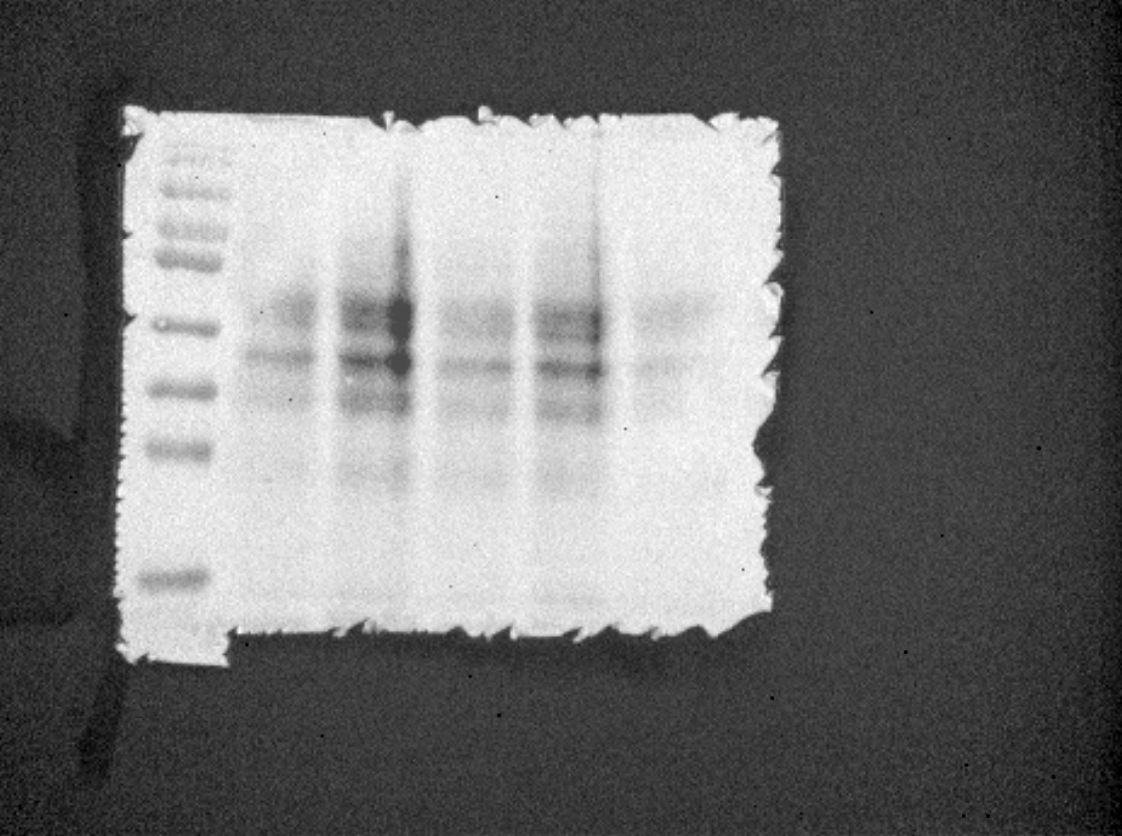

Supplement: Supplementary file 6 — High Resolution Image (TIF 2.68 mb) [file 11011_2025_1634_MOESM3_ESM.tif]
